# Post-OGTT hypoglycaemia in newly diagnosed type 2 diabetes: frequency, characteristics and cluster assignment

**DOI:** 10.1007/s00125-026-06719-0

**Published:** 2026-03-27

**Authors:** Rebecca Spies, Gisa Ufer, Roza Sabia, Julia Hummel, Andreas Peter, Robert Wagner, Martin Heni

**Affiliations:** 1 Division of Endocrinology and Diabetology, Department of Internal Medicine I, University Hospital Ulm, Ulm, Germany; 2 Institute for Clinical Chemistry and Pathobiochemistry, Department for Diagnostic Laboratory Medicine, University Hospital Tübingen, Tübingen, Germany; 3 Institute for Diabetes Research and Metabolic Diseases of the Helmholtz Center Munich at the University of Tübingen, Tübingen, Germany; 4 German Center for Diabetes Research (DZD), Neuherberg, Germany; 5 Division of Endocrinology and Diabetology, Medical Faculty, Heinrich Heine University, Düsseldorf, Germany; 6 Institute for Clinical Diabetology, German Diabetes Center, Leibniz Institute for Diabetes Research at Heinrich Heine University, Düsseldorf, Germany

**Keywords:** Diabetes subtypes, Insulin sensitivity, Oral glucose tolerance test, Post-OGTT hypoglycaemia, Reactive hypoglycaemia, Type 2 diabetes

## Abstract

**Aims/hypothesis:**

The aim of this study was to assess the frequency and metabolic characteristics of post-OGTT hypoglycaemia during an OGTT in individuals newly diagnosed with type 2 diabetes.

**Methods:**

We analysed 97 extended (180 min) 75 g OGTTs from individuals newly diagnosed with type 2 diabetes. Glucose, insulin and C-peptide were measured at fasting and post-challenge time points. Insulin sensitivity was assessed using the oral glucose insulin sensitivity (OGIS) index and beta cell function was measured using the insulinogenic index (IGI) and Stumvoll’s second-phase index. All participants were assigned to the Ahlqvist diabetes clusters.

**Results:**

Post-OGTT hypoglycaemia (glucose ≤3.9 mmol/l at 180 min) occurred in 8.2% of the OGTTs analysed. Compared with individuals without hypoglycaemia, individuals with post-OGTT hypoglycaemia had a significantly lower BMI and higher insulin sensitivity, without differences in beta cell function. All individuals with hypoglycaemia were assigned to the mild obesity- or age-related diabetes Ahlqvist clusters.

**Conclusions/interpretation:**

Post-OGTT hypoglycaemia is present in a subset of individuals newly diagnosed with type 2 diabetes and is associated with higher insulin sensitivity.

**Graphical Abstract:**

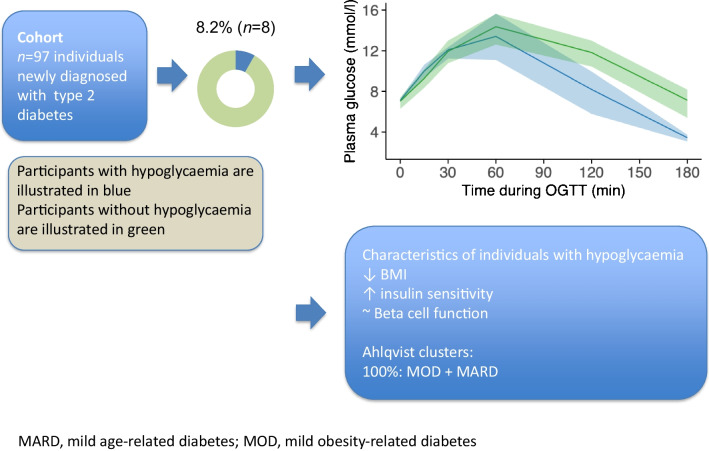


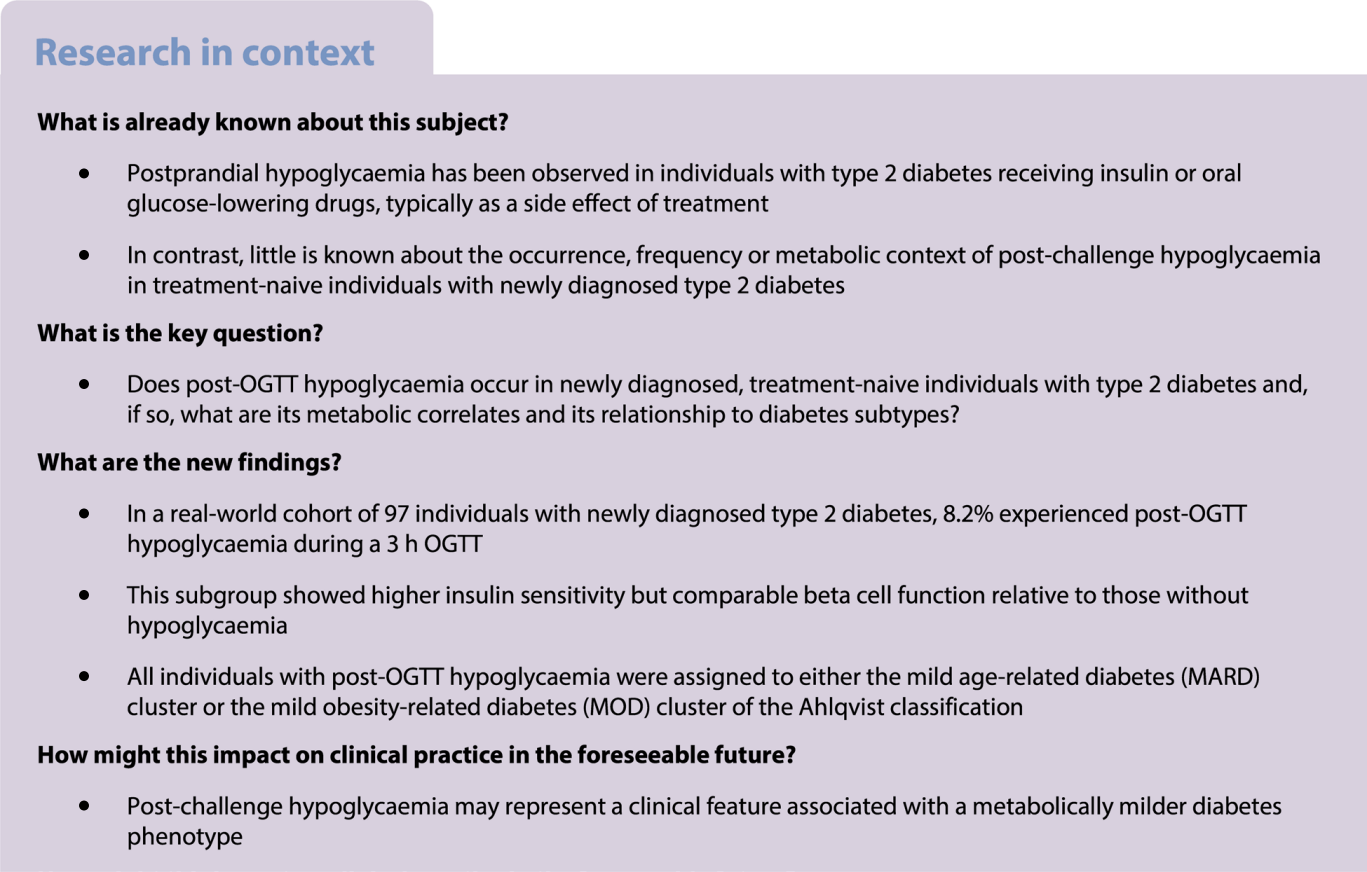


## Introduction

Postprandial hypoglycaemia refers to a significant drop in blood glucose after, for example, a carbohydrate-rich meal. While traditionally attributed at least in part to excessive insulin secretion, other mechanisms independent of insulin secretion have also been proposed [1–3]. Whether postprandial or post-OGTT hypoglycaemia occurs in individuals with newly diagnosed type 2 diabetes remains unclear. Moreover, the underlying mechanisms involved, such as excessive insulin secretion or enhanced insulin sensitivity, have not been systematically studied in this context. To address these gaps, we analysed data from extended 180 min OGTTs in which type 2 diabetes was newly diagnosed, with the aim of assessing the frequency, metabolic characteristics and potential drivers of post-OGTT hypoglycaemia in early type 2 diabetes.

## Methods

We analysed data from 97 extended OGTTs in which type 2 diabetes was newly diagnosed. None of the participants was taking glucose-lowering medications at the time of the OGTT. The tests were conducted between 2012 and 2023 at University Hospital Ulm as part of routine medical care. Therefore, only individuals with an indication for an OGTT, based on the treating physician’s judgement, were included. All available OGTTs with complete datasets were included; there were no further exclusions. Sex was self-reported. After an overnight fast, a prolonged 75 g OGTT was performed, with venous blood samples collected before and at 15, 30, 60, 120 and 180 min after glucose ingestion. Plasma glucose, insulin, C-peptide and additional routine parameters were measured in the central laboratory of University Hospital Ulm. Beta cell function was assessed using the insulinogenic index (IGI) and Stumvoll’s second-phase insulin secretion index [4]; insulin sensitivity was assessed using HOMA-IR [5] and the oral glucose insulin sensitivity (OGIS) index [6]. This retrospective analysis was approved by the local ethics committee of Ulm University (approval number 411/22). All participants provided informed written consent. Statistical analyses were conducted using R (version 2024.12.0 + 467, Posit Software) [7]. Comparisons between the hypoglycaemia and control groups were performed using Wilcoxon rank-sum tests. Adjustment for BMI was conducted using a rank-based ANCOVA. The significance level was set at *p*<0.05. Additionally, participants were clustered using the online DDZ Diabetes Cluster Tool (https://diabetescalculator.ddz.de/diabetescluster/), which allocates individuals to the five Ahlqvist diabetes clusters [8, 9]. Cluster distribution between groups was tested using Fisher’s exact test.

## Results

Among the analysed OGTTs, which were selected based on a new diagnosis of type 2 diabetes, diabetes was diagnosed based on 2 h plasma glucose in 37.1% of participants, on elevated fasting plasma glucose in 30.9% of participants, and on a combination of both in 24.5% of participants. Only 7.2% of participants had an HbA_1c_ >48 mmol/mol (>6.5%). Of the 97 individuals, 46 were female (47.4%). Participant characteristics are presented in Table 1.

**Table 1:** Characteristics of the 97 individuals with newly diagnosed type 2 diabetes

Characteristic	Post-OGTT hypoglycaemia (*n*=8)	No hypoglycaemia (*n=*89)	*p* value^a^	*p* value, BMI adjusted^b^
Sex (female)	6 (75)	40 (45)	0.21	–
Age (years)	58 (56–65)	54 (44–60)	0.11	0.15
BMI (kg/m^2^)	29.36 (26.42–32.12)	32.74 (29.00–39.93)	0.06	–
eGFR (ml/min per 1.73 m^2^)	86 (78–91)	88 (72–100)	0.52	0.53
Fasting glucose (mmol/l)	7.19 (7.02–7.26)	7.08 (6.26–7.38)	0.72	0.75
Glucose, 120 min (mmol/l)	6.91 (6.05–8.56)	11.85 (10.46–12.97)	0.004	0.002
Glucose, 180 min (mmol/l)	3.55 (3.05–3.73)	6.83 (5.34–8.16)	<0.001	<0.001
HbA_1c_ (mmol/l)	44.3 (43.4–44.3)	42.1 (38.8–45.3)	0.32	
HbA_1c_ (%)	6.2 (6.1–6.2)	6.0 (5.7–6.3)	0.32	0.25
HOMA-IR (AU)	5.71 (4.30–7.65)	7.75 (5.08–12.81)	0.04	0.08
OGIS index (AU)	324 (288–364)	266 (222–314)	0.04	0.04
IGI (AU)	0.71 (0.51–1.10)	0.70 (0.47–1.06)	0.70	0.75
Stumvoll’s second-phase index (AU)	500 (368–567)	493 (331–695)	0.87	0.95

Data are presented as median (IQR) or *n* (%)

The eight individuals in the hypoglycaemia group had a blood glucose concentration ≤3.9 mmol/l at 180 min during an OGTT, at which type 2 diabetes was newly diagnosed

^a^Group comparisons were performed using the χ^2^ test (sex) and Wilcoxon rank-sum test

^b^BMI-adjusted *p* values were obtained using a rank-based ANCOVA

AU, arbitrary units

**Fig. 1:**
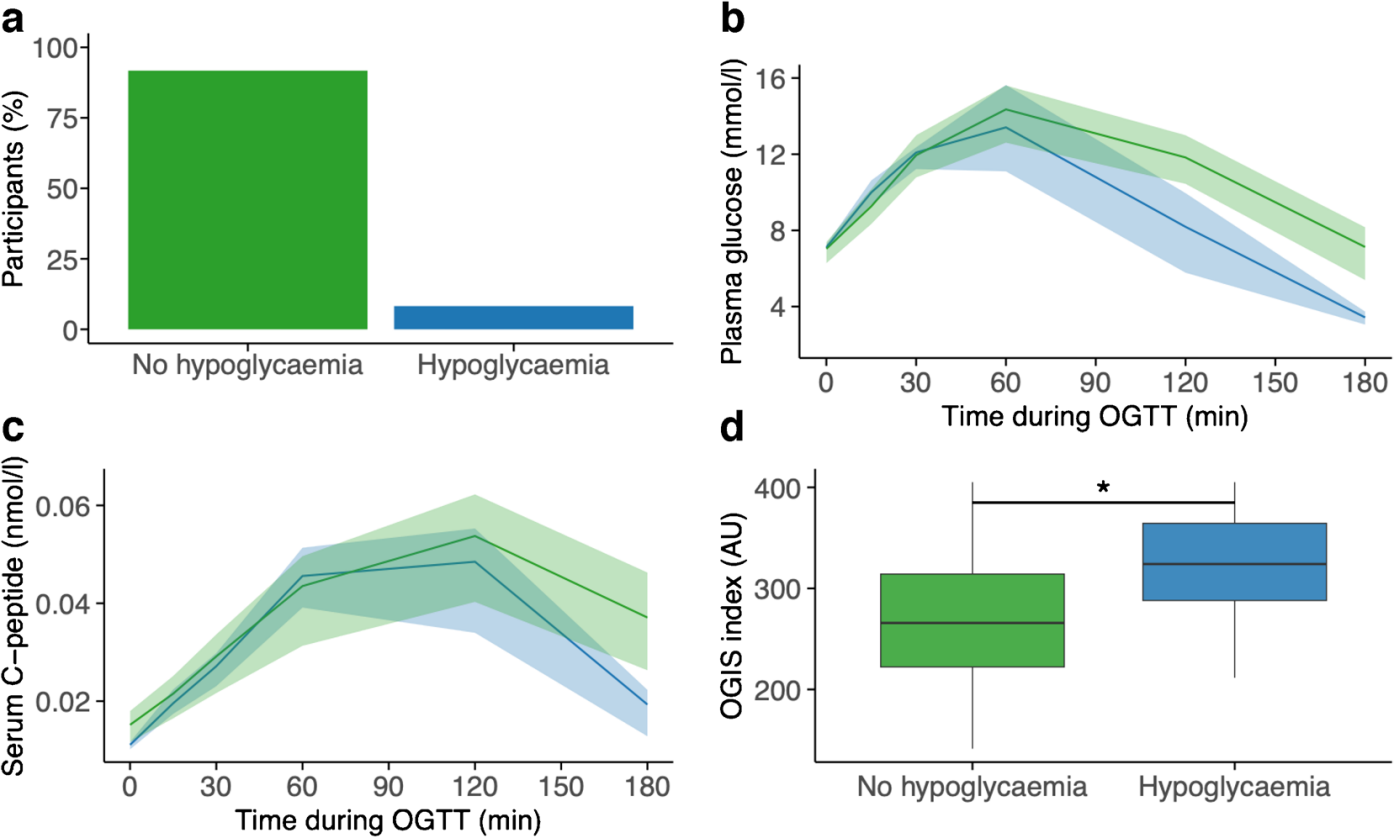
(**a**) Distribution of the 97 individuals with newly diagnosed type 2 diabetes based on the presence or absence of hypoglycaemia at 180 min during a 75 g OGTT. Hypoglycaemia was defined as glucose ≤3.9 mmol/l at 180 min. (**b**) Plasma glucose and (**c**) serum C-peptide during a 75 g OGTT in individuals with newly diagnosed type 2 diabetes. (**d**) OGIS index in those with and without hypoglycaemia at 180 min during a 75 g OGTT. *p<0.05. (**b**, **c**) Data are presented as median (IQR). (**d**) Horizontal lines represent the median and boxes indicate the IQR. Whiskers extend to values within 1.5 × IQR from the lower and upper quartiles. Blue curves/bars indicate individuals with post-OGTT hypoglycaemia (glucose ≤3.9 mmol/l at 180 min); green curves/bars indicate individuals without hypoglycaemia. AU, arbitrary units

At 180 min post load, hypoglycaemia (plasma glucose concentration ≤3.9 mmol/l) occurred in eight of the included participants (8.2%) (Fig. 1a), with one of these individuals (1%) exhibiting a plasma glucose concentration ≤3.0 mmol/l (Fig. 1b). In the hypoglycaemia group, six of eight individuals were female (75%), compared with 45% in the group without hypoglycaemia. C-peptide levels were not higher in those who experienced hypoglycaemia (Fig. 1c).

Individuals with post-OGTT hypoglycaemia had a lower BMI (median [IQR] 29.36 [26.42–32.12] vs 32.74 [29.00–39.93] kg/m^2^; *p*=0.06) and tended to be older (58 [56–65] vs 53.5 [44–60] years; *p*=0.11) than individuals without hypoglycaemia. Insulin sensitivity was higher in those with post-OGTT hypoglycaemia than in those without (assessed using HOMA-IR: *p*=0.04; OGIS index: *p*=0.04) (Fig. 1d), whereas indices of beta cell function did not differ between groups (assessed using IGI: *p*=0.70; Stumvoll’s second-phase index: *p*=0.87).

To further characterise these individuals, all participants were assigned to the five Ahlqvist diabetes clusters [8, 9]. The distribution of these clusters differed between individuals with and individuals without post-OGTT hypoglycaemia (*p*=0.04). All individuals with post-OGTT hypoglycaemia were assigned to the milder Ahlqvist clusters: five to mild age-related diabetes (MARD) and three to mild obesity-related diabetes (MOD). Among those without hypoglycaemia, 70% also belonged to these milder clusters (50% MOD, 19% MARD), while 30% were assigned to the severe insulin-resistant diabetes (SIRD) cluster. Only 1% were classified as having severe insulin-deficient diabetes (SIDD). Thus, post-OGTT hypoglycaemia occurred exclusively within the milder clusters in our cohort but was not specific to them. However, comparing the number of participants in the more severe clusters (SIRD and SIDD combined) with those in the milder clusters (MARD and MOD combined) did not reveal statistically significant differences (*p*=0.1).

## Discussion

Post-OGTT hypoglycaemia was observed in a subset of individuals with newly diagnosed type 2 diabetes during an extended OGTT. In contrast to the traditional assumption that excessive insulin secretion is the primary mechanism involved, our findings support the hypothesis that this phenomenon is associated with higher insulin sensitivity. Interestingly, early insulin secretion loss in prediabetes has been linked to delayed but excessive insulin release, which may contribute to post-OGTT hypoglycaemia [3, 10]. However, we did not observe consistent differences across multiple indices of beta cell function, making this explanation unlikely in our cohort.

The clinical implications of post-OGTT hypoglycaemia in early type 2 diabetes remain unclear. Whether it represents a distinct, more insulin-sensitive metabolic subtype or indicates altered disease progression warrants further investigation. Notably, all individuals with post-OGTT hypoglycaemia were assigned to either the MARD or the MOD Ahlqvist cluster. These clusters are typically associated with slower disease progression, fewer metabolic complications and relatively preserved beta cell function compared with more severe diabetes subtypes [9, 11]. However, as a substantial proportion of individuals without hypoglycaemia also belonged to these clusters, post-OGTT hypoglycaemia should be interpreted as an enriched feature within milder diabetes phenotypes rather than a defining characteristic. Future studies should assess whether post-OGTT hypoglycaemia predicts long-term glycaemic management and whether hypoglycaemia also occurs under real-life conditions, outside standardised testing.

Limitations of our study include the modest sample size and the fact that all participants were recruited from an outpatient clinic of a university hospital, which may limit generalisability. Additionally, no gold standard tests for insulin sensitivity (hyperinsulinaemic–euglycaemic glucose clamp) or insulin secretion (hyperglycaemic clamp/i.v. glucose tolerance test) were performed, and no incretin measurements were available, limiting mechanistic conclusions.

Overall, our findings indicate that post-OGTT hypoglycaemia is present in a subset of individuals with newly diagnosed type 2 diabetes and associates with higher insulin sensitivity.

## Data Availability

The data generated during the current study are not publicly available because of privacy and ethical restrictions.

## Abbreviations

IGI: Insulinogenic index
OGIS: Oral glucose insulin sensitivity
MARD: Mild age-related diabetes
MOD: Mild obesity-related diabetes
SIDD: Severe insulin-deficient diabetes
SIRD: Severe insulin-resistant diabetes

## Authors’ relationships and activities

Outside the current work, RW reports honoraria during the past 36 months for lectures/presentations/speaker’s bureaus from Daiichi Sankyo, Eli Lilly, Boehringer Ingelheim, Novo Nordisk, Sanofi-Aventis and Synlab; travel support from Eli Lilly, Novo Nordisk, Daiichi Sankyo and Sanofi-Aventis; and honoraria for advisory boards from Eli Lilly, Boehringer Ingelheim and Sanofi-Aventis. GU reports lecture fees from Boehringer Ingelheim and travel support from Ipsen, Lilly and Boehringer Ingelheim. Outside the current work, RSa reports presentations/speaker’s fees from Amgen. She has also served on advisory boards for Sobi, Recordati Rare Diseases and Novartis. MH reports lecture fees from Amryt/Chiesi, AstraZeneca, Bayer, Boehringer Ingelheim, Daiichi Sankyo, Lilly, Novartis, Novo Nordisk and Sanofi-Aventis. He has also served on advisory boards for Amryt/Chiesi and Boehringer Ingelheim. The authors declare that there are no other relationships or activities that might bias, or be perceived to bias, their work.
